# Health literacy, dietary behavior and body mass index in male and female Norwegian conscripts. A cross-sectional study

**DOI:** 10.1177/22799036261461038

**Published:** 2026-07-08

**Authors:** Nima Wesseltoft-Rao, Elisabeth Rudjord Hillesund, Nina Cecilie Øverby, Heming Olsen-Bergem, Kristine Vejrup

**Affiliations:** 1 Faculty of Health Studies, Institute of Nursing, 87368 VID Specialized University, Oslo, Norway; 2 Faculty of Health and Sports Sciences, Department of Nutrition and Public Health, 4343 University of Agder, Kristiansand, Norway; 3 Institute for Military Epidemiology, 587219 The Norwegian Armed Forces Joint Medical Services, Sessvollmoen, Norway; 4 Department of Oral Surgery and Oral Medicine, Faculty of Dentistry, 69622 University of Oslo, Oslo, Norway

**Keywords:** health literacy, armed forces, conscripts, dietary habits, body mass index/BMI

## Abstract

**Objectives:**

Health literacy (HL) is an important determinant of health behaviors, including diet. In military populations, optimal nutrition is critical for physical performance, readiness, and long-term health. In Norway, first-time military service provides a unique opportunity to influence young conscripts’ health habits. This study investigates the relationship between HL and dietary patterns, body mass index (BMI), and meal adherence among Norwegian conscripts, with an emphasis on potential sex differences.

**Study Design:**

A cross-sectional study.

**Methods:**

This cross-sectional study included Norwegian conscripts completing first-time military service. HL was measured using the validated HLS-Q12 instrument. Dietary behaviors, meal patterns, and BMI were assessed using self-reported questionnaire data. A digital questionnaire was sent to Norwegian conscripts completing their first-time military service in 2022 (n=9,991). A total of 2,225 conscripts consented to participate in the study. The survey assessed dietary habits across different military settings, self-reported diet quality, and HL using the HLS-Q12 tool. BMI was calculated from self-reported height and weight. Statistical analyses included chi-square tests and Pearson correlation to explore associations between HL, diet, and BMI.

**Results:**

Significant associations were observed between HL and several dietary variables; however, most associations were weak in magnitude. Higher HL was associated with more regular meal patterns and better self-reported diet quality. Additionally, differences between sexes were observed, but effect sizes were generally small. More than half of conscripts had an adequate HL, with males scoring significantly higher than females. Conscripts with adequate HL were more likely to rate their diet as good, follow meal recommendations, and maintain a normal BMI. However, no significant association between HL and BMI was found in the total population; differences appeared in sex-stratified analyses, among female conscripts. Independently of HL, unhealthy snacking and high-sugar drink consumption were more common during field training, whereas conscripts with inadequate HL (iHL) reporting higher sugary snack intake at home. Adequate HL was weakly but significantly correlated with higher self-reported diet quality, more frequent adherence to regular meals, and lower sugary snack consumption. This could mean that HL was associated with self-reported dietary intentions but not outcomes like BMI.

**Conclusions:**

HL may be associated with dietary behaviors among conscripts. However, given the weak associations and study limitations, the findings should be interpreted with caution. Although more than half of the conscripts had adequate HL, iHL was prevalent among Norwegian conscripts. Poorer self-reported diet quality and lower adherence to regular meal patterns is associated with iHL, particularly during field training. No significant associations was found between HL and BMI. The findings suggest a need for tailored education to improve HL and behavior change, to promote healthier dietary habits during military service.

## Introduction

Health literacy (HL) refers to the ability to access, understand, appraise, and apply health information.^1^ Poor HL is associated with non-communicable diseases and limited HL has been linked to diet-related and metabolic health outcomes.^2–4^ Dietary patterns high in vegetables, fruits, whole grains, fish, low-fat dairy and legumes, and low in red and processed meats, sugar-sweetened beverages, sugary foods- and refined grains are associated with decreased risk of chronic diseases,^5,6^ and indicate a high diet quality.^7^ There are no specific recommendations for meal patterns,^6^ however three-four main meals per day, and one-two between-meal snacks per day can be a way to ensure stable energy throughout the day and secure the intake of all necessary nutrients.^8,9^

Overweight (body mass index (BMI) > 25.0-29.9 kg/m^2^) and obesity (BMI> 30 kg/m^2^) are known risk factors for reduced quality of life and non-communicable diseases (NCDs), and contribute to rising global healthcare costs.^10^ Preventing overweight and obesity in young people will yield greater societal and individual health benefits through life.^11^ Prevention strategies, such as implementing policies to reduce obesogenic environments, as well as promoting healthy diet and physical activity, are crucial to mitigating the epidemic of overweight and obesity.^12,13^

Previous research has shown that young conscripts often exhibit suboptimal dietary habits. A 2022 pilot study from Norway, found that many conscripts had unfavorable dietary patterns, with nearly half reporting daily consumption of sweets/chocolate or sugary drinks.^14^ In military settings, recruits often experience a transition in eating environments, which may influence both diet quality and meal regularity. In military populations, dietary behaviors are particularly important due to their direct impact on physical performance.^15^ Promoting HL and healthy eating behaviors within military service could be crucial in order to contribute to good health throughout a lifetime.^16^

There is no defined recommended level of HL, but international research and public health authorities generally suggest that a high proportion of the population (ideally 80–85%) should have adequate HL to effectively understand health information, make informed health decisions, and navigate health care systems.^17^ Baseline levels of HL and dietary preferences differ between young men and women and may have impact upon their adaptation to military life and routines.^18,19^ Recent international studies found that women often choose healthier foods and eat regular meals, while men show preferences for specific tastes and meal-related behaviors, such as eating quickly and eating out.^20^ Meals served in the cantinas in military camps are designed for high-caloric requirement and physical endurance in mind and may not appeal to preferences of both sexes.^21^

Although HL has been widely studied in civilian populations, fewer studies have examined its relationship with dietary behaviors in military conscripts. The Norwegian first-time military service is comparable in some respects to basic recruits in other military systems.^22,23^ Moreover, nutrition policy and education structures differ across militaries internationally. Understanding how diet and meal patterns relates to HL in first-time military service in Norway, alongside potential sex differences, can provide valuable insights into the management of tailored health education programs that support conscripts’ health, resilience, and long-term well-being. The aim of this study was therefore to examine associations between HL, dietary patterns, and BMI among Norwegian conscripts, with a focus on sex differences.

## Methods

### Study population

A digital questionnaire was sent to conscripts who completed first-time military service in Norway in November-December 2022. A total of n= 9,991 individuals were invited to participate, n=2,225 (22%) responded and consented to their data being used in research. There were no exclusion criteria in this study. A total of n=1,858 (18%) participants completed all 12 questions in the HLS-Q12. Totally the survey had a response rate of 22%, which is at the lower end of the acceptable range. However, there were no signs of systematic bias. Since this was an observational study, the results are valuable in order to do further measures to map HL among young conscripts.

### The questionnaire

The questionnaire was developed collaboratively by The Arctic University of Norway, the University of Oslo, and the Norwegian Armed Forces Health Register (NAFHR). It consisted of 38 questions covering oral hygiene, tobacco use, and diet across different military settings (field exercises, in camp, and at home). HL was assessed using the validated European Health Literacy Survey Questionnaire-Q12 (HLS-Q12). The HLS-Q12 is a validated short-form instrument derived from the original 47-item European Health Literacy Survey Questionnaire (HLS-EU-Q47). It was developed to provide a concise yet psychometrically robust measure of general HL across the domains of accessing, understanding, appraising, and applying health information.^14^ Details about the questionnaire are reported elsewhere.^22^

### Ethics

This survey was part of an internal quality study of the Norwegian Armed Forces’ (NAF)' routines, aiming to identify potential need for improving routines during the initial training of conscripts. The project was assessed by the Norwegian Regional Ethical Committee (reference number: 806000) and approved by the NAF Joint Medical Services.

### Data included in the study

Responses with unreasonable measures were excluded (n=9 for height <140 cm or >220 cm, and n=6 for weight <40 kg or >200 kg). BMI was calculated for participants who reported both height and weight and categorized according to WHO definitions: BMI <18.5: underweight. BMI 18.5-24.9: normal weight. BMI ≥25.0: overweight. BMI ≥30.0: obesity.

### Assessment of health literacy

The questionnaire HLS-Q12 is shown in Supplemental Table 1. The questionnaire provided HL scores (HLS) ranging from 12 to 48, with HLS of ≥33 indicating adequate HL in line with scoring methods. “Don’t know” responses in the HLS scale were re-coded as missing data to maintain consistency with established scoring methods. Participants who did not answer one or more questions, or who answered, “don’t know”, achieved a score below 12. Participants with HLS below 12 were also re-coded as missing, in line with scoring protocol.^14^ Participants were grouped into two categories according to HLS, with 12 to 32 representing inadequate health literacy (iHL) (coded as 1), and from 33-48 represent adequate health literacy (coded as 2).^14^ Grouping of the variables was done in order to simplify statistical analyses.

A STROBE checklist was used.

### Dietary assessment

The questionnaire addressed meal patterns, self-reported diet quality, snacking habits, and beverage consumption across three military settings: field exercises, in camp, and at home.

Conscripts recorded the frequency of eating meals (breakfast, lunch, dinner, late supper) per week at home and in camp, with response options ranging from “never” to “every day.” Meal patterns were categorized as: 3–4 meals/day = 1 or <3 meals/day = 0.

Conscripts rated their diet quality on a 5-point Likert scale (1 = very low, 5 = very high), subsequently grouped into three categories: Low (very low and low) =1, average =2, and high (high and very high) = 3.

Conscripts reported the frequency of consuming different types of snacks, such as candy, chocolate, salty snacks, nuts, protein bars, fruit and yogurt, re-coded into categories ranging from “never” to “more than 6 times/day.” The snacks and convenience foods, including protein bars, were analyzed without categorizing them strictly as healthy or unhealthy**.**

Beverage consumption was assessed across military settings and in connection with meals, with the options: water, squash, juice, coffee and tea, energy drinks and soft drinks. High-sugar beverages were defined as containing ≥50g of sugar per 500 ml. These sugary drinks are available to buy in designated shops on camps. Frequency of consumption of sugary drinks was re-coded into categories ranging from “never” to “more than 6 times/day.”

### Statistics

The dataset was stratified on sex and HLS categories to explore group differences. Descriptive statistics were used to assess differences in diet quality, BMI, and meal patterns.

The Pearson Chi-square test was used to determine significant differences in diet quality, BMI, and meal patterns between participants with adequate and inadequate HLS, both overall and stratified by sex. Pearson correlations analysis was performed between HLS, diet quality, sex and dietary habits.

## Results

The study population (n=2,225) consisted of 54% males and 46% females, with a mean age of 20 years (range: 19–27). The majority (60%) had a normal weight, while 18% were classified as overweight (data not shown). Less than 1% of the conscripts were classified as underweight, and 2% were obese. About 19% had missing reasonable height or weight. Males had a higher mean height and weight; thus higher BMI compared to females. Overweight was slightly more prevalent among males compared to women (26% vs 18%) (Table 1).

**Table 1.** Descriptive table of age, height, weight, BMI, health literacy (HL), and self-rated dietary quality, stratified by sex.

​	Total n=2,225	Female N=1,015	Male N=1,210	P-values
**Age (mean, range)**	20 (19-27)	20 (19-24)	20 (19-27)	N/A
**Height (cm, mean, range)**	176 (151-203)	168 (151-187)	183 (153-203)	<0.001
**Weight (kg, mean, range)**	74 (43-180)	66 (43-173)	81 (50-180)	<0.001
**BMI (kg/m** ^**2**^ **, mean, range)**	24 (16-46)	23 (16-55)	24 (17-46)	<0.001
**BMI categories (%)**				
underweight (BMI <18.5) n (%)	10 (1)	7 (1)	3 (1)	​
normal weight (BMI 18.5-24.9) n (%)	1332 (74)	647 (79)	685 (71)	​
overweight (BMI 25.0-29.9) n (%)	402 (23)	150 (18)	252 (26)	​
obesity (BMI ≥30.0) n (%)	36 (2)	12 (2)	24 (2)	​
Total*	1780	816	964	​
**HL**	​	​	​	<0.001
inadequate HL n (%)	773 (43)	413 (49)	360 (37)	​
Adequate HL n (%)	1036 (57)	422 (51)	614 (63)	​
Total*	1809	835	974	​
**Self-rated diet quality** n (%)	​	​	​	<0.001
very poor	11 (1)	4 (1)	7 (1)	​
poor	118 (6)	54 (6)	64 (6)	​
adequate	653 (35)	318 (37)	335 (33)	​
good	937 (51)	439 (52)	498 (50)	​
very good	133 (7)	37 (4)	96 (10)	​
Total*	1852	852	1000	​

*missing BMI n=445; *missing HLS-Q12 n=416; *missing Self-rated diet quality n=373.

A total of 1,858 participants completed all 12 questions in the HLS-Q12. The missing group (n=416) consisted of 367 participants who did not answer the HLS-Q12 and 49 participants with a score below 12. About 57% had an adequate HL, with a significantly higher proportion of males (63%) scoring adequately compared to females (51%).

Around 51% rated their diet as “good,” with a higher proportion of females rating their diet as “adequate” or “good” compared to males. About 55% reported eating three or more meals per day in military camp setting, but adhering to the regular meal pattern was lower at home (46%). Reported snacking frequency varied by setting; During field exercises, conscripts more frequently consumed packed snacks such as candy, chocolate, and protein bars. Healthier options like fruit and yogurt were rarely consumed. In camp, snacking was more moderate, with higher fruit and yogurt consumption compared to field exercises. Nearly half of the conscripts still reported daily intake of snacks, candy, and chocolate. At home, snacking frequency was lower across most food categories, with a slight reduction in unhealthy snack consumption compared to camp. Beverage consumption differed by setting. Energy drinks and sugary drinks were most frequently consumed during field exercises, followed by coffee/tea, juice, and sugar-sweetened soft drinks. Beverage consumption in camp and at home, was mainly water. Juice was more frequently consumed in camps, while milk and both sugary and sugar-free sodas were preferred at home. Coffee/tea and energy drink consumption showed minimal differences between settings.

When comparing self-rated diet quality according to HLS, a significant difference was observed. The association between HL and self-reported diet quality was highly collinear. Conscripts with adequate HL were more likely to rate their diet as “good” (66%) compared to those with iHL (47%) (Table 2). This trend was consistent across sexes.

**Table 2.** Comparison of Self-Rated Diet Quality, BMI, and Meal Patterns by Health Literacy scoring (HLS), stratified by sex.

HLS-Q12 score	Total study population (n=1809)		Male (n= 974)		Female (n= 835)	
	inadequate (n=773)	adequate (n=1036)	inadequate (n=360)	adequate (n=614)	inadequate (n=413)	adequate (n=422)
**Self-rated diet**						
bad	73 (9)	52 (5)	35 (10)	35 (6)	38 (9)	17 (4)
adequate	336 (44)	296 (29)	156 (43)	166 (27)	180 (44)	130 (31)
good	361 (47)	658 (66)	169 (47)	412 (67)	192 (47)	273 (65)
**BMI**						
underweight (BMI<18.5)	5 (1)	4 (1)	1 (1)	2 (1)	4 (1)	2 (1)
normal weight (BMI 18.5-24.9)	532 (72)	765 (77)	237 (68)	429 (73)	295 (75)	336 (83)
overweight (BMI ≥25.0)	183 (25)	209 (21)	100 (29)	145 (25)	83 (21)	64 (16)
obesity (BMI ≥30.0)	20 (3)	16 (2)	10 (3)	14 (2)	10 (3)	2 (1)
**meal pattern**						
Field exercises						
<3 meals per day	302 (39)	353 (34)	131 (36)	193 (31)	171 (41)	160 (38)
3- 4 meals per day	471 (61)	683 (66)	229 (64)	421 (69)	242 (59)	262 (62)
Camp						
<3 meals per day	365 (47)	435 (42)	154 (43)	253 (41)	211 (51)	182 (43)
3- 4 meals per day	408 (53)	601 (58)	206 (57)	361 (59)	202 (49)	240(57)
Home						
<3 meals per day	455 (59)	518 (50)	193 (54)	276 (45)	262 (63)	242 (57)
3- 4 meals per day	318 (41)	518 (50)	167 (46)	338 (55)	151 (37)	180 (43)

Sex-stratified analyses showed that BMI distributions differed between males and females across both HL groups (Table 2). Overall, sex-based differences in BMI were present in both HL groups but were more pronounced in the iHL category. In the iHL group, males had a higher proportion of overweight compared to females (29% vs. 21%), while obesity prevalence was similar (3% in both sexes). A comparable pattern was observed in the adequate HL group, where males also had a higher proportion of overweight than females (25% vs. 16%), and slightly higher obesity prevalence (2% vs. 1%). Regular meal patterns were more often seen in conscripts with adequate HLS, particularly in camps and during field exercises. Sex differences were evident, with males consistently showing more regular meal patterns and diet quality ratings than females. The association between HL and meal patterns was observed across settings. In sex-stratified analyses, HL was significantly associated with meal patterns in camp among females, whereas for males, this association was observed at home.

Regarding high-sugar drinks and snack consumption, a significant difference was found only for sugary snack intake at home, where participants with iHL had higher consumption. When stratified by sex, this difference remained significant only for male conscripts (data not shown). All data are available upon request.

When performing Pearson correlation analysis, several significant crude associations emerged between HLS and other variables. These associations were statistically significant but weak in magnitude.

HL was positively correlated with self-rated diet quality with a weak correlation (r ≈ 0.21, p < 0.001), and a similarly weak positive relationship was observed with sex (r ≈ 0.13, p < 0.001). Additionally, HL showed very weak positive correlations with the frequency of consuming regular meals in all military settings; field (r ≈ 0.05, p = 0.041), camp (r ≈ 0.05, p = 0.025), and home (r ≈ 0.11, p < 0.001). There was also a very weak positive correlation between HL and frequency of eating sugary snacks at home (r ≈ 0.06, p = 0.018).

In parallel, self-rated diet quality demonstrated a weak negative association with BMI category (r ≈ –0.14, p < 0.001), indicating that better diet quality is related to a lower BMI. Self-rated diet quality was also positively associated with consumption of regular meals—most notably in the home setting (r ≈ 0.23, p < 0.001) and in camp (r ≈ 0.19, p < 0.001)—while being negatively correlated with high-sugar drink intake, particularly at home (r ≈ –0.18, p < 0.001) and in camp (r ≈ –0.15, p < 0.001).

Overall, although these associations are statistically significant, their strengths are modest, suggesting that the relationships between HL, self-rated diet quality, and the associated behavioral factors are reliable yet weak in magnitude selection bias.

## Discussion

In this study, 57% of the conscripts had adequate HL, and 43% had iHL, as defined by Lillegård et al.^14^ Conscripts with higher HL reported healthier dietary behaviors, however no associations was found between HL and BMI. In exercises and out-of-camp settings conscripts reported lower nutritional awareness regardless of HL levels.

A balanced diet is crucial for combat abilities, especially in a prolonged military conflict.^23^ Armies may benefit from adjusting nutrition education, and food provision for conscripts based on their HL, to secure healthy soldiers in peace, and adaptable soldiers in war.^24^

Our findings suggest that the relationship between HL and eating behavior observed in the general population holds true in a military context,^25^ whereas HL influences nutrition practices such as understanding food labels to choose healthy food, which in turn improves dietary quality.^4,26^ Conscripts with high HL might be more adept at understanding nutrition information, leading to healthier overall diets.^26^

HL was only weakly associated with BMI in our study, which is in line with earlier research.^27^ Studies report that HL is directly associated with physical activity, but is only indirectly associated with BMI,^17,28^ suggesting that HLs effect on weight status might be indirect or only observable over a longer term. Furthermore, several other factors than HL, such as peer pressure, cultural- and physiological factors, or stress coping behaviors in the field might affect diet and BMI regardless of HL.^22,29–31^ Other research emphasizes the importance of healthy eating patterns, such as not skipping meals, for weight control.^32^ This implies that if HL encourages more regular meal routines, it could eventually translate into healthier body weight.^15^

Differences between males and females were observed. However, these differences were generally small and should be interpreted cautiously. Regarding sex differences, women in both civilian and military samples often exhibit healthier eating behaviors than men.^20^ In a comparable military context, British Army recruits of both sexes were found to under-consume certain nutrients, but men consumed significantly more energy, often from less nutrient-dense sources, than women.^33^ Female recruits tend to favor fruits, vegetables and other healthy options more than male recruits, who report higher intakes of red meat and fast food.^20^ Our findings that Norwegian female conscripts had lower HL and reported a better diet quality then men, may indicate that there is difference in how men and women self-report their HL, similar to another study showing that men tended to overestimate their HL.^34^

Our study’s relationships between HL, self-rated diet quality, and other dietary habits align with prior research on HL and nutrition both in civilian young adults and in military personnel while also highlighting the unique environmental challenges of the military setting.^35,36^

Although the data in our study are self-reported, and have weak associations, they reveal a potential for improving behaviors and outcomes if tailored education is provided to build capability and opportunity, enact behavior and generate motivation.^37^ Dietary habits can be shaped through structured routines, regular meals, and physical conditioning. These factors can influence conscripts HL and impact their dietary choices both during and after service.^38,39^ The findings carry important implications for both military readiness and public health.^15,36^ First, the high prevalence of iHL among conscripts (nearly one in two) is concerning from a public health perspective. Targeted education during service can strengthen HL in this population and may empower conscripts to make better nutritional choices, thereby contributing to long-term public health benefits.^40^ To address the high prevalence of iHL and associated dietary habits, the NAF may consider implementing targeted interventions during first-time service. These could include the integration of a HL module into basic training, focusing on nutrition, hydration, label reading, and meal planning, as suggested by Fallowfield et al.^24^ Also, introducing a mobile-friendly self-monitoring tool, integrated with existing military systems, to help recruits track meals, hydration, and energy levels could promote informed food choices. Our findings support that field-adapted nutrition guidance and ration packs to include more balanced, nutrient-dense options could aid decisions during exercises.^15,41^ We also identify an opportunity for the NAF to collaborate with the Norwegian national health authorities to align military HL strategies with broader youth-focused health initiatives.

The findings from our study are broadly consistent with studies in civilian populations^42^ but add to the limited literature in military conscripts.^38^ Furthermore, differences in military systems and nutrition policies limit direct comparison across countries.

The strengths of this study is the large sample size and the use of a validated HL instrument. Several limitations of this study should be acknowledged when interpreting the results.

First, the data were self-reported, which is prone to measurement bias such as under-reporting of unhealthy food intake or overestimating healthy behaviors, and recall and desirability bias. The self-reported data may reflect intentions of behavior rather than actual behavior. The analytical sample differed from the total invited population due to non-response and incomplete data, which may affect representativeness of the data. Second, cross-sectional design does not allow inference of causality. Confounding factors such as education level and family background could not be controlled for and may have explained part of the HL-diet relationship. Additionally, the sex differences may be explained by the fact that female conscripts are a self-selected subgroup who may differ in motivation or health consciousness from the general population of women.

Third, the HLS-Q12, is a shortened questionnaire. While it is a validated instrument for general HL, it provides a broad overview rather than detailed insights into nutrition-specific literacy. Some conscripts classified as having “adequate” HL may still lack specific nutrition knowledge. Fourth, the study relied on a one-time survey conducted while conscripts were at different stages of their first-time service. The seasonal timing of questionnaire distribution may have influenced responses and is acknowledged as a limitation. The differences we found between field and home settings may be under- or over-estimated if participants didn’t accurately recall their behaviors during field exercises. Finally, as a pilot study and an internal quality improvement survey, there may be some degree of selection bias in the findings related to who chose to respond. Low response rate may lead to potential selection bias. The response rate of 22% means that almost ¾ of the conscripts did not participate. A low response rate can be acceptable when; the population is homogeneous (e.g. male and female conscripts in a closed setting and population, represented by the first time military service); The responses are consistent across the group; there was no obvious systematic bias across the group. Finally, the topic was not very controversial, so that the motivation to respond didn’t vary greatly. The soldiers who actually completed the survey may be of the compliant type, or they may have considered the survey topic to be more important or interesting than those who did not respond. This may contribute to a less representative sample. If non-responders systematically have different HL or poorer dietary habits, our results could paint a somewhat overly positive picture. Other limitations of the study were; no a priori sample size calculation was performed because the questionnaire was distributed to the full eligible population as part of a pilot study; the weak effect sizes indicating that HL alone may not be a strong determinant of dietary behavior in this population; the diet quality was not based on a validated index; there were possible collinearities between variables, and finally seasonal and contextual variation not accounted for. The results should therefore be interpreted as indicative rather than conclusive. Future studies can build on this groundwork with more robust designs to address these caveats. Emphasizing health benefits, body image appearance, and increased energy and fitness can motivate young people to adopt healthier lifestyles.^43^ Furthermore, addressing the barriers for behavioral change, such as accessibility of unhealthy foods, can nudge young people into healthier behaviors.^13^

### Conclusion

HL was associated with dietary behaviors among Norwegian conscripts.

These findings indicate important implications for interventions. However, given the weak associations and study limitations, the findings should be interpreted with caution. Although more than half of the Norwegian conscripts had adequate HL, the level of iHL was prevalent in this group. The level of iHL was associated with poorer self-rated diet quality and lower adherence to regular meal patterns, particularly during field training. No strong link between HL and BMI was observed in the total group. The findings suggest a need to improve HL and promote healthier dietary habits during military service for targeted interventions.

Military service time can act as a channel for reaching young adults during a critical transition to independence. By systematically addressing the gaps in nutrition knowledge and the environmental hurdles identified in this study, the NAF can set young people on a path toward better nutrition and HL that will benefit them during military service and far beyond. Future longitudinal and intervention-based studies are warranted to explore causal pathways and test preventive strategies. Future research should explore causal pathways and evaluate interventions targeting both HL and structural determinants of diet in military settings.

## Supplemental material

Supplemental material - Health literacy, dietary behavior and body mass index in male and female Norwegian conscripts. A cross-sectional study Supplemental material for Health literacy, dietary behavior and body mass index in male and female Norwegian conscripts. A cross-sectional study by Nima Wesseltoft-Rao, Elisabet Rudjord Hillesund, Nina Cecilie Øverby, Heming Olsen-Bergem, Kristine Vejrup in Journal of Public Health Research

## Data Availability

The data are available on request from the NAFHR. All data is freely accessible provided that required approvals are available.*
