# Supplementary material for: Health literacy, dietary behavior and body mass index in male and female Norwegian conscripts. A cross-sectional study
Source: J Public Health Res. 2026 Jul 8;15(3):22799036261461038. doi: 10.1177/22799036261461038 (PMC13346719; doi:10.1177/22799036261461038)
Supplement: Supplemental material - Health literacy, dietary behavior and body mass index in male and female Norwegian conscripts. A cross-sectional study [file sj-pdf-1-phj-10.1177_22799036261461038.pdf]

Supplementary table 1. The European Health Literacy Questionnaire short version HLS-Q12.

On a scale from very difficult to very easy, how easy would you say it is to:

	Very hard	Hard	Easy	Very easy	Don't know
Find information about treatments for a disease that applies to you?					
Understand what to do in a medical emergency?					
Assess the advantages and disadvantages of different types of treatment?					
Follow the instructions for use stated on the packaging of medicines?					
Find information on how psychological problems such as stress and depression can be dealt with?					
Understand why you need general health examinations (e.g. mammography, measuring blood sugar and blood pressure)?					
Assess whether information provided by the media about health risks can be trusted (TV, internet or other media)?					
Decide how you can avoid illness on the basis of advice from family and friends?					
Find information about healthy habits, such as exercise, healthy food and proper nutrition?					
Understand information on food packaging?					
Assess which everyday habits are related to your health (eating and drinking habits, exercise, etc.)?					
Make decisions to improve your health?					

KAN OFFENTLIGGJØRES iht. Sikkerhetsdirektivet pkt. 4.2
